# A non-invasive, home-based biomechanical therapy for patients with spontaneous osteonecrosis of the knee

**DOI:** 10.1186/s13018-016-0472-0

**Published:** 2016-11-14

**Authors:** Ehud Atoun, Amit Mor, Ganit Segal, Ronen Debi, Dan Grinberg, Yeshaiau Benedict, Nimrod Rozen, Yiftah Beer, Avi Elbaz

**Affiliations:** 1 Department of Orthopedic Surgery, Barzilai Medical Center, Ashkelon, Israel; 2 AposTherapy Research Group, 1 Abba Even Blvd., 46733 Herzliya, Israel; 3 Department of Orthopedic Surgery, HaEmek Medical Center, Afula, Israel; 4 Department of Orthopedic Surgery, Assaf Harofeh Medical Center, Zerifin, Israel

**Keywords:** SONK, Biomechanical treatment, Pain, Function

## Abstract

**Background:**

The purpose of the current study was to examine the effect of a non-invasive, home-based biomechanical treatment program for patients with spontaneous osteonecrosis of the knee (SONK).

**Methods:**

Seventeen patients with SONK, confirmed by MRI, participated in this retrospective analysis. Patients underwent a spatiotemporal gait analysis and completed the Western Ontario and McMaster University Osteoarthritis Index (WOMAC) and the Short-Form-36 (SF-36). Following an initial assessment, patients commenced the biomechanical treatment (AposTherapy). All patients were reassessed after 3 and 6 months of treatment.

**Results:**

A significant reduction in pain and improvement in function was seen after 3 months of therapy with additional improvement after 6 months of therapy. Pain was reduced by 53% and functional limitation reduced by 43%. Furthermore, a significant improvement was also found in the SF-36 subscales, including the summary of physical and mental scores. Significant improvements were found in most of the gait parameters including a 41% increase in gait velocity and a 22% increase in step length. Patients also demonstrated improvement in limb symmetry, especially by increasing the single limb support of the involved limb.

**Conclusions:**

Applying this therapy allowed patients to be active, while walking more symmetrically and with less pain. With time, the natural course of the disease alongside the activity of the patients with the unique biomechanical device led to a significant reduction in pain and improved gait patterns. Therefore, we believe AposTherapy should be considered as a treatment option for patients with SONK.

**Trial registration:**

Assaf Harofeh Medical Center Institutional Helsinki Committee Registry, 141/08; ClinicalTrials.gov NCT00767780.

## Background

The knee, after the hip, is the second most common site for osteonecrosis (ON) [1]. Spontaneous osteonecrosis of the knee (SONK), first described by Ahlback et al. [2] in 1968, is considered to be the most common form of ON, with an incidence of 3.4 and 9.4% in persons older than 50 and 65 years of age, respectively [3]. However, the actual prevalence may be underestimated since many patients with end-stage osteoarthritis (OA) may have had an undiagnosed occult condition [4].

SONK is classically described as a focal, superficial subchondral lesion, affecting the medial femoral condyle in up to 94% of the time [5, 6]. The presenting symptom is usually an acute onset of pain over the medial side of the knee [7]. Focal tenderness over the medial femoral condyle is the most common finding on physical examination [8]. Patients often present deteriorated, asymmetrical gait patterns [9] and complain that the pain is worse during weight-bearing and at night [4]. The etiology of SONK remains unclear. Historically, it was thought to occur secondary to ischemia, which results in necrosis [2, 10]. However, recent evidence has demonstrated that it may be due to subchondral insufficiency fractures in osteopenic bone with no evidence of necrosis [5].

Non-operative management of SONK includes treatment with non-steroidal anti-inflammatory drugs (NSAIDs), protected weight-bearing, analgesics, high-dose of vitamin D supplementation, and bisphosphonate [11]. Surgical management includes joint-preserving techniques such as arthroscopic debridement and core decompression [12–14]. In end-stage SONK, uni-compartmental knee arthroplasty or total knee arthroplasty are the most common treatment options [15, 16]. Surprisingly, although SONK is a fairly common, severely disabling, and frequently deteriorating condition, there is only a paucity of studies describing the different treatment alternatives and no randomized or high-quality studies comparing different treatment options have been described [7, 11, 17–19].

In the last half decade, several publications have described the effect of treatment with a unique non-invasive, home-based biomechanical therapy on clinical symptoms and gait patterns of patients with different musculoskeletal conditions including knee pathologies, such as knee OA [20–22], degenerative meniscal tear [23], and anterior knee pain [24]. The aim of the current study was to examine the effect of this non-invasive biomechanical treatment on gait patterns and clinical symptoms of patients with SONK.

## Methods

### Patients

This was a retrospective analysis based on a private clinic’s database. The protocol of the current study was similar to previous publications of our research group [23, 24], hence the similarity in the research methodology. However, for the first time, this research work focused on patients with SONK. The protocol was approved by Assaf Harofeh Medical Center Institutional Helsinki Committee Registry (Helsinki registration number 141/08, NIH protocol no. NCT00767780). Since this was a retrospective study, the ethics committee waived the need for individual consent forms.

A search for eligible patients diagnosed with SONK and confirmed by MRI was done on the clinic’s database from January 2010 to August 2015. Inclusion criteria for the study were SONK confirmed by MRI and having gait data and questionnaires at pre-treatment assessment and after 3 and 6 months of therapy. Exclusion criteria included a history of major trauma, predisposing factors of osteonecrosis, previous surgery to the knee excluding arthroscopy, and knee arthroscopy in the 3 months prior to the first assessment. Eighty-seven patients diagnosed with SONK commenced therapy during the abovementioned period. Seventy patients were excluded since they did not meet the inclusion criteria and/or had one or more of the exclusion criteria. A total of 17 patients were included in the analysis; their characteristics are presented in Table 1.

**Table 1:** Patients’ characteristics

	Mean (SD)	Range
Male/female (%)	7/10 (41/59)	
Age (years)	65.2 (9.7)	41–85
Weight (kg)	83.1 (13.0)	58–105
Height (cm)	162.6 (11.3)	142–183
Duration of symptoms (months)	6.2 (5.5)	1–24

### Assessments

All patients underwent a spatiotemporal gait assessment and completed clinical questionnaires to assess pain, function, and quality of life at pre-treatment assessment and following 3 and 6 months of therapy.

*Anamnesis.* During their first visit to the therapy center, patients underwent systematic assessment including a physical examination by a certified physical therapist and anthropometric measurements of height and weight.

*Spatiotemporal gait assessment.* Using a computerized mat (GaitMat system, E.Q., Inc. Chalfont, PA) [25], patients were asked to walk barefoot at a self-selected speed. Patients walked 3 m before and after the walkway mat to allow sufficient acceleration and deceleration time outside the measurement area. Four trials were conducted, and acquired data were stored for further analysis. The mean value of the four trials was calculated for each of the following parameters: velocity (cm/s), step length (cm), cadence (steps/min), base of support (BOS) (cm), swing (% gait cycle (GC)), stance (% GC), single limb support (% GC) (SLS), and double limb support (% GC) (DLS). Where applicable, results are presented for the involved limb and the uninvolved limb.

*Clinical outcomes*. Patients completed the Western Ontario and McMaster Osteoarthritis Index (WOMAC) to assess pain and function. This questionnaire contains 24 questions using a visual analogue scale (VAS). Results may range from 0 to 100 mm, with 0 mm indicating no pain, stiffness, or limitation in function and 100 mm indicating the most severe pain, stiffness, or limitation in function. The mean average of the 24 questions creates an overall score. In addition, three subscales are calculated: 5 questions to assess pain, 2 questions to assess joint stiffness, and 17 questions to assess function.

Patients also completed the Short-Form (SF)-36 Health Survey to assess the quality of life (QoL). This questionnaire contains 36 Likert scale questions regarding different aspects of QoL. The SF-36 is scored between 0 and 100, with 0 indicating the worst quality of life and 100 indicating the best quality of life. An overall score is calculated from the results of all questions. Furthermore, eight subscales can also be calculated including physical functioning, pain, limitation due to physical health, vitality, emotional well-being, limitation due to mental health, social functioning, and general health. Two summarizing scores are also available: a physical component summary (PCS) which is the average score of the following four categories: physical functioning, pain, limitation due to physical health, and general health; and a mental component summary (MCS) which is the average score of the following four categories: vitality, emotional well-being, limitation due to mental health, and social functioning.

### Intervention

**Fig. 1:**
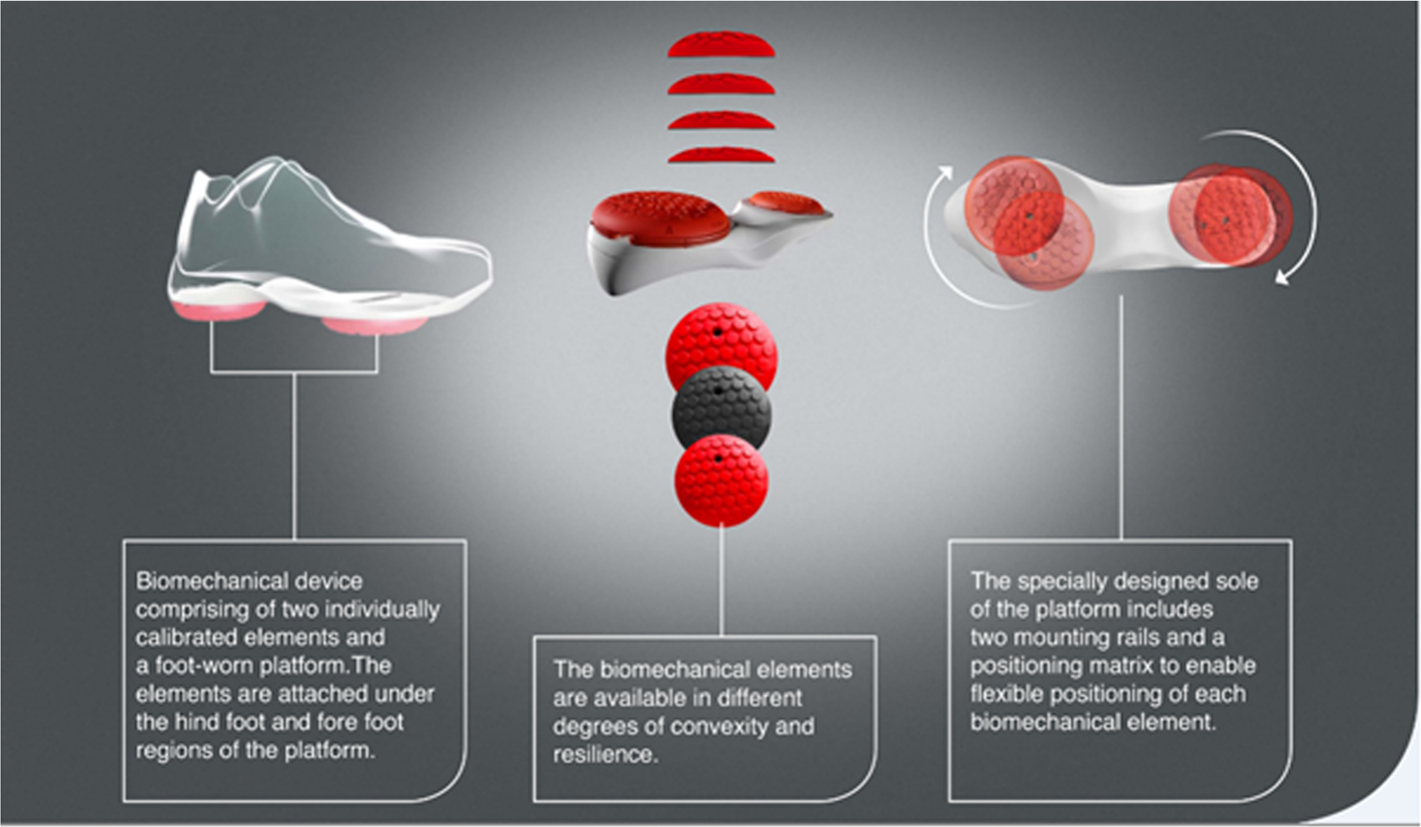
AposSystem. Biomechanical device

The biomechanical device (Apos System, Apos–Medical and Sports Technologies Ltd. Herzliya, Israel) utilized in the study and the treatment modality have been previously described [20, 22, 26, 27]. In brief, the device consists of two convex-shaped biomechanical elements attached to each foot using a platform in the form of a shoe, allowing customized calibration (Fig. 1). By shifting the biomechanical elements in the coronal and sagittal planes, the device can be individually calibrated to shift the trajectory of the foot’s center of pressure during gait, thereby altering the orientation of the ground reaction force vector. This enables a decrease in the pressure load from the affected area in the joint during gait [28–34]. The convex form of the biomechanical elements generates perturbations applied throughout the stance phase of the gait cycle [35], enabling dynamic, functional, and repetitive training intended to improve neuromuscular control. Following enrolment, the biomechanical device was individually calibrated to each patient by a licensed physical therapist specialized in AposTherapy methodology. Treatment was then initiated and continued on a daily basis for a period of 6 months. Patients were instructed to wear the biomechanical device for 10 min once a day during the first week, while performing daily routine (accumulating 5 min of walk). Patients were instructed to gradually increase walking time reaching 60 min once a day (accumulating 30 min of walk).

### Statistical analysis

All spatiotemporal gait parameters and self-evaluation questionnaires scores were presented as mean and standard deviation, followed by 95% confidence interval for all time periods. Non-parametric one-sample Kolmogorov-Smirnov tests were calculated to compare the observed cumulative distribution function for the continuous variables with the normal theoretical distribution. The GLM Repeated Measures procedures and Friedman non-parametric tests were used to provide level of improvement for gait parameters and self-evaluation questionnaires when the same measurement was made three times on each subject.

The comparison between the involved and uninvolved limb was conducted where applicable using paired *t* tests.

The correlations between the changes in gait velocity (from pre-treatment assessment to 6 months’ follow-up) and the changes in pain and function (from pre-treatment assessment to 6 months’ follow-up) were assessed using Spearman correlations.

Data were analyzed with IBM SPSS software version 23.0, and the significant level was set at 0.05.

## Results

All patients complied with the treatment and completed the study protocol with no adverse events reported. Significant improvement was found in all gait measures except for the base of support, stance phase of the involved limb, swing phase of the involved limb, and SLS phase of the uninvolved limb (Table 2). Furthermore, a comparison between the involved and uninvolved limb was also conducted where applicable. At pre-treatment assessment, significant differences were found between the involved and uninvolved limb in the following parameters: swing (*p* < 0.001), stance (*p* < 0.001), and SLS (*p* < 0.001). After 3 months of treatment, significant differences between limbs were found in swing (*p* = 0.028), stance (*p* = 0.028), and SLS (*p* = 0.009). After 6 months of treatment, significant differences between limbs were found in swing (*p* = 0.011), stance (*p* = 0.011), and SLS (*p* = 0.009).

**Table 2:** Changes in spatiotemporal gait following 6 months of treatment. Results are presented as mean (SD) [95% confidence interval, CI]

	Pre-treatment	3 months	6 months	*P* for GLM test	*P* for Friedman test
Velocity (cm/s)	65.2 (25.6) [52.0–78.3]	85.4 (23.8) [73.2–97.7]	92.1 (23.4) [80.1–104.2]	*P* < 0.001	*P* < 0.001
Cadence (steps/min)	64.1 (14.0) [56.8–71.3]	73.9 (13.9) [66.7–81.0]	76.4 (13.6) [69.5–83.4]	*P* < 0.001	*P* < 0.001
Base of support (cm)	7.5 (3.7) [5.6–9.4]	6.7 (2.6) [5.3–8.0]	6.3 (2.8) [4.8–7.7]	*P* = 0.104	*P* = 0.257
Step length—involved (cm)	43.6 (11.5) [37.7–49.5]	50.9 (11.5) [45.0–56.8]	53.4 (12.6) [46.9–59.9]	*P* < 0.001	*P* < 0.001
Step length—uninvolved (cm)	44.7 (13.5) [37.7–51.6]	51.2 (12.7) [44.7–57.7]	53.9 (11.9) [47.8–60.0]	*P* < 0.001	*P* < 0.001
Stance—involved (% GC)	61.8 (3.6) [60.0–63.6]	62.3 (2.5) [61.0–63.6]	61.9 (2.5) [60.6–63.2]	*P* = 0.875	*P* = 0.101
Stance—uninvolved (% GC)	68.3 (4.7) [65.9–70.8]	64.0 (3.7) [62.1–65.9]	63.2 (2.9) [61.8–64.7]	*P* < 0.001	*P* < 0.001
Swing—involved (% GC)	38.2 (3.6) [36.4–40.0]	37.7 (2.5) [36.4–39.0]	38.1 (2.5) 36.8–39.4]	*P* = 0.875	*P* = 0.101
Swing—uninvolved (% GC)	31.7 (4.7) [29.2–34.1]	36.0 (3.7) [34.1–37.9]	36.8 (2.9) [35.3–38.2]	*P* < 0.001	*P* < 0.001
SLS—involved (% GC)	31.8 (4.6) [29.5–34.2]	35.9 (3.6) [34.0–37.8]	36.8 (2.8) [35.4–38.3]	*P* < 0.001	*P* < 0.001
SLS—uninvolved (% GC)	38.3 (3.6) [36.5–40.2]	37.8 (2.6) [36.5–39.2]	38.2 (2.4) [36.9–39.4]	*P* = 0.858	*P* = 0.589
DLS—involved (% GC)	30 (5.9) [26.9–33.0]	26.4 (5.7) [23.4–39.3]	25.1 (5.1) [22.5–27.7]	*P* < 0.001	*P* < 0.001
DLS—uninvolved (% GC)	30 (6.0) [26.9–33.1]	26.2 (5.8) [23.2–29.2]	25.1 (5.0) [22.5–27.6]	*P* < 0.001	*P* < 0.001

*GC* gait cycle, *SLS* single limb support, *DLS* double limb support

**Fig. 2:**
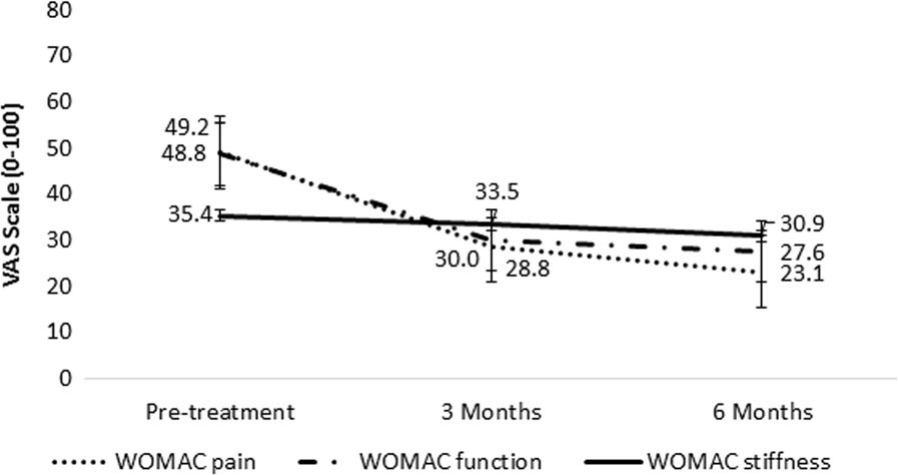
Changes in WOMAC subscales following 6 months of treatment

Significant improvements were also found in the clinical outcomes of pain, function, and QoL. Changes in WOMAC subscales are presented in Fig. 2. Alongside the statistical significance, patients also met the OMERACT-OARSI clinical criteria for clinical significance [36]. Changes in SF-36 overall score, subscales, and PCS and MCS are presented in Table 3. Patients met the minimal clinical important difference (MCID) for rehabilitation intervention for patients with osteoarthritis of the lower extremity [37].

**Table 3:** Changes in SF-36 subscales following 6 months of treatment. Results are presented as mean (SD) [95 % confidence interval, CI]

	Pre-treatment	3 months	6 months	*P* for GLM test	*P* for Friedman test
Overall score	41.6 (16.6) [33.1–50.1]	52.4 (17.5) [43.4–61.4]	54.5 (22.5) [42.9–66.1]	*P* = 0.017	*P* = 0.003
Physical functioning	36.5 (28.2) [22.0–51.0]	49.7 (21.1) [38.9–60.6]	53.2 (27.9) [38.9–67.6]	*P* = 0.032	*P* = 0.032
Pain	32.5 (23.5) [20.4–44.6]	54.6 (24.0) [42.2–66.9]	58.8 (27.5) [44.7–73.0]	*P* = 0.002	*P* = 0.006
Limitation due to physical health	22.1 (36.3) [3.4–40.7]	39.7 (41.5) [18.4–61.1]	41.2 (46.7) [17.2–65.2]	*P* = 0.097	*P* = 0.008
Vitality	48.5 (14.2) [41.2–55.8]	53.8 (14.0) [46.6–61.0]	51.5 (18.4) [42.0–60.9]	*P* = 0.543	*P* = 0.049
Emotional well-being	54.4 (17.0) [45.6–63.1]	62.6 (14.6) [55.1–70.1]	63.5 (18.7) [53.9–73.1]	*P* = 0.023	*P* = 0.129
Limitation due to mental health	29.4 (42.3) [7.7–51.2]	39.2 (41.2) [18.0–60.4]	49.0 (48.8) [24.0–74.1]	*P* = 0.136	*P* = 0.414
Social functioning	46.3 (21.5) [35.2–57.4]	66.2 (22.0) [54.9–77.5]	65.4 (26.0) [52.1–78.8]	*P* = 0.010	*P* = 0.002
General health	55.6 (14.4) [48.2–63.1]	57.4 (15.3) [49.5–65.2]	57.8 (17.0) [49.1–66.6]	*P* = 0.570	*P* = 0.678
PCS	36.7 (19.4) [26.7–46.6]	50.3 (21.2) [39.4–61.2]	52.8 (26.1) [39.4–66.2]	*P* = 0.017	*P* = 0.005
MCS	44.7 (17.5) [35.7–53.6]	55.5 (18.6) [45.9–65.0]	57.4 (23.9) [45.1–69.7]	*P* = 0.026	*P* = 0.028

*PCS* physical component summary, *MCS* mental component summary

The correlations between the changes in gait velocity (from pre-treatment assessment to 6 months’ follow-up) and the changes in pain and function (from pre-treatment assessment to 6 months’ follow-up) were calculated. A significant moderate correlation was found between the changes in gait velocity and the changes in pain (*r* = −0.535, *p* = 0.027). The correlation between changes in gait velocity and changes in function was −0.553 (*p* = 0.021).

## Discussion

The purpose of the current study was to examine the effect of this non-invasive biomechanical treatment on gait patterns and clinical symptoms of patients with SONK. Following 6 months of treatment, a significant improvement in gait pattern and quality of life and a significant reduction in pain was noted.

The knee is a weight-bearing joint which contends with massive loads during locomotion. Previous studies have shown that gait patterns are compromised as a result of different musculoskeletal conditions in general and specifically in knee conditions [38–41]. A recent study showed that patients with SONK present altered gait patterns compared to healthy individuals [9]. These alterations may be due to a new gait strategy adopted by the patients in order to avoid joint loading and pain. Furthermore, a major deviation in gait patterns of patients with SONK is asymmetry in selected gait parameters including single limb support, which reflects the ability of the patient to bear loads on one limb while the contralateral limb swings forward. This ability decreases dramatically in the involved limb and marked asymmetry is present in patients with SONK. This is a decisive factor since biomechanical asymmetries may have long-term consequences, providing further support for the potential role of loading on the onset and progression of knee osteoarthritis [42]. The results of the current study show that after 3 months of treatment with a unique biomechanical device, there was a significant improvement in gait which continued to improve after 6 months. Patients presented an overall increase of 41% in gait velocity, 22% in step length, and 19% in cadence. The improvement in velocity is crucial as it has been linked to survival in older adults [43]. Patients also improved their ability to bear loads on the affected limb, reflecting limb symmetry. Although significant differences between limbs in SLS were still present at 6 months, there was a substantial reduction in limb asymmetry. It may be assumed that should this study have continued with a longer follow-up, an additional improvement including the elimination of significant asymmetry would have been found. We believe that this therapy allowed patients *to walk the extra mile*, mainly by enabling them to be active while implementing less compensations by walking more symmetrically.

Although gait is an objective tool to assess the functional condition of patients and results can be compared between patients, it is also important to assess the clinical changes over time with regard to pain, function, and QoL. There was a moderate correlation between the improvement in gait velocity and the improvement in pain and function. Patients were assessed using gold standard questionnaires and reported a significant improvement in pain (53%), function (43%), PCS (44%), and MCS (28%), meeting the criteria for clinical significance [36, 37]. Furthermore, the effect size of the change was calculated and was found to be high for pain and function and medium for PCS and MCS [44, 45]. Effect sizes for pain, function, PCS, and MCS were 1.05, 0.92, 0.70, and 0.60, respectively.

To the best of our knowledge, this is the first time a non-pharmacological, non-invasive treatment is offered to patients with SONK. This treatment is based on walking with a biomechanical device and allows patients to be active. The unique structure of the device, which can change the center of pressure and thereby change the loads at the knee joint, enables patients to walk with reduced pain while improving neuromuscular control. The current study showed positive results for patients with SONK, adding support to the positive effect of treatment for other knee conditions including degenerative meniscal tear [23], ACL tear [46], and anterior knee pain [24].

Some limitations should be acknowledged. First, this was a single cohort study with no control group. The lack of a control group makes it difficult to conclude that the treatment is better than other alternatives. However, this study presents a positive trend and should be considered as an additional non-invasive treatment option for patients with SONK. It should be emphasized that none of the patients required any surgical intervention during the follow-up period. We acknowledge that further research is necessary and recommend that a future study should examine the effect of treatment for patients with SONK in a randomized controlled trial. This will support the preliminary results of the current study. Second, this was a retrospective analysis of patients seeking treatment at a private clinic. As such, the study population may have been biased to those who were exposed to this clinic rather than the entire population. We postulate that this had a minor effect on the results and that the group’s characteristics are good representatives of the population. Third, this study monitored the changes in objective gait patterns and clinical outcomes. Having a randomized controlled trial with an additional MRI assessment of the involved knee after 6 months would have given a clearer picture of the changes in the knee joint over time.

## Conclusions

Following 6 months of therapy, patients presented a significant improvement in gait and gained a more symmetrical gait pattern. Patients also reported a significant reduction in pain and improvement in function and QoL, meeting the gold standard criteria for clinical significance.

## Abbreviations

BOS: Base of support
DLS: Double limb support
GC: Gait cycle
MCS: Mental component summary
NSAIDs: Non-steroidal anti-inflammatory drugs
OA: Osteoarthritis
ON: Osteonecrosis
PCS: Physical component summary
QoL: Quality of life
SF: Short-Form
SLS: Single limb support
SONK: Spontaneous osteonecrosis of the knee
VAS: Visual analogue scale
WOMAC: Western Ontario and McMaster Osteoarthritis Index

## References

[1] Mont MA, Baumgarten KM, Rifai A, Bluemke DA, Jones LC, Hungerford DS (2000). Atraumatic osteonecrosis of the knee. J Bone Joint Surg Am.

[2] Ahlback S, Bauer GC, Bohne WH (1968). Spontaneous osteonecrosis of the knee. Arthritis Rheum.

[3] Pape D, Seil R, Fritsch E, Rupp S, Kohn D (2002). Prevalence of spontaneous osteonecrosis of the medial femoral condyle in elderly patients. Knee Surg Sports Traumatol Arthrosc.

[4] Mont MA, Marker DR, Zywiel MG, Carrino JA (2011). Osteonecrosis of the knee and related conditions. J Am Acad Orthop Surg.

[5] Yamamoto T, Bullough PG (2000). Spontaneous osteonecrosis of the knee: the result of subchondral insufficiency fracture. J Bone Joint Surg Am.

[6] al-Rowaih A, Bjorkengren A, Egund N, Lindstrand A, Wingstrand H, Thorngren KG (1993). Size of osteonecrosis of the knee. Clin Orthop Relat Res.

[7] Karim AR, Cherian JJ, Jauregui JJ, Pierce T, Mont MA (2015). Osteonecrosis of the knee: review. Ann Transl Med.

[8] Houpt JB, Pritzker KP, Alpert B, Greyson ND, Gross AE (1983). Natural history of spontaneous osteonecrosis of the knee (SONK): a review. Semin Arthritis Rheum.

[9] Atoun E, Segal G, Debi R, Lubovsky O, Djabbarov R, Peskin B (2016). Gait assessment of patients with spontaneous osteonecrosis of the knee. Osteoarthritis and Cartilage.

[10] Aglietti P, Insall JN, Buzzi R, Deschamps G (1983). Idiopathic osteonecrosis of the knee. Aetiology, prognosis and treatment. J Bone Joint Surg Br.

[11] Jureus J, Lindstrand A, Geijer M, Robertsson O, Tagil M (2013). The natural course of spontaneous osteonecrosis of the knee (SPONK): a 1- to 27-year follow-up of 40 patients. Acta Orthop.

[12] Miller GK, Maylahn DJ, Drennan DB (1986). The treatment of idiopathic osteonecrosis of the medial femoral condyle with arthroscopic debridement. Arthroscopy.

[13] Akgun I, Kesmezacar H, Ogut T, Kebudi A, Kanberoglu K (2005). Arthroscopic microfracture treatment for osteonecrosis of the knee. Arthroscopy.

[14] Forst J, Forst R, Heller KD, Adam G (1998). Spontaneous osteonecrosis of the femoral condyle: causal treatment by early core decompression. Arch Orthop Trauma Surg.

[15] Heyse TJ, Khefacha A, Fuchs-Winkelmann S, Cartier P (2011). UKA after spontaneous osteonecrosis of the knee: a retrospective analysis. Arch Orthop Trauma Surg.

[16] Myers TG, Cui Q, Kuskowski M, Mihalko WM, Saleh KJ (2006). Outcomes of total and unicompartmental knee arthroplasty for secondary and spontaneous osteonecrosis of the knee. J Bone Joint Surg Am..

[17] Breer S, Oheim R, Krause M, Marshall RP, Amling M, Barvencik F (2013). Spontaneous osteonecrosis of the knee (SONK). Knee Surg Sports Traumatol Arthrosc.

[18] Kraenzlin ME, Graf C, Meier C, Kraenzlin C, Friedrich NF (2010). Possible beneficial effect of bisphosphonates in osteonecrosis of the knee. Knee Surg Sports Traumatol Arthrosc.

[19] Jureus J, Lindstrand A, Geijer M, Roberts D, Tagil M (2012). Treatment of spontaneous osteonecrosis of the knee (SPONK) by a bisphosphonate. Acta Orthop.

[20] Bar-Ziv Y, Beer Y, Ran Y, Benedict S, Halperin N (2010). A treatment applying a biomechanical device to the feet of patients with knee osteoarthritis results in reduced pain and improved function: a prospective controlled study. BMC Musculoskelet Disord..

[21] Elbaz A, Mor A, Segal G, Debbi E, Haim A, Halperin N (2010). APOS therapy improves clinical measurements and gait in patients with knee osteoarthritis. Clin Biomech (Bristol, Avon).

[22] Haim A, Rubin G, Rozen N, Goryachev Y, Wolf A (2012). Reduction in knee adduction moment via non-invasive biomechanical training: a longitudinal gait analysis study. J Biomech.

[23] Elbaz A, Beer Y, Rath E, Morag G, Segal G, Debbi EM (2013). A unique foot-worn device for patients with degenerative meniscal tear. Knee Surg Sports Traumatol Arthrosc.

[24] Haim A, Segal G, Elbaz A, Mor A, Agar G, Bar-Ziv Y (2013). The outcome of a novel biomechanical therapy for patients suffering from anterior knee pain. Knee.

[25] Barker S, Craik R, Freedman W, Herrmann N, Hillstrom H (2006). Accuracy, reliability, and validity of a spatiotemporal gait analysis system. Med Eng Phys.

[26] Debbi EM, Wolf A, Goryachev Y, Rozen N, Haim A (2015). Alterations in sagittal plane knee kinetics in knee osteoarthritis using a biomechanical therapy device. Ann Biomed Eng.

[27] Elbaz A, Mor A, Segal G, Aloni Y, Teo TH, Teo YS (2014). Patients with knee osteoarthritis demonstrate improved gait pattern and reduced pain following a non-invasive biomechanical therapy: a prospective multi-center study on Singaporean population. J Orthop Surg Res..

[28] Haim A, Rozen N, Dekel S, Halperin N, Wolf A (2008). Control of knee coronal plane moment via modulation of center of pressure: a prospective gait analysis study. J Biomech.

[29] Haim A, Rozen N, Wolf A (2010). The influence of sagittal center of pressure offset on gait kinematics and kinetics. J Biomech.

[30] Haim A, Wolf A, Rubin G, Genis Y, Khoury M, Rozen N (2011). Effect of center of pressure modulation on knee adduction moment in medial compartment knee osteoarthritis. J Orthop Res.

[31] Khoury M, Wolf A, Debbi EM, Herman A, Haim A (2013). Foot center of pressure trajectory alteration by biomechanical manipulation of shoe design. Foot Ankle Int.

[32] Khoury M, Haim A, Herman A, Rozen N, Wolf A (2015). Alteration of the foot center of pressure trajectory by an unstable shoe design. J Foot Ankle Res..

[33] Solomonow-Avnon D, Wolf A, Herman A, Rozen N, Haim A (2015). Reduction of frontal-plane hip joint reaction force via medio-lateral foot center of pressure manipulation: a pilot study. J Orthop Res.

[34] Solomonow-Avnon D, Haim A, Levin D, Elboim-Gabyzon M, Rozen N, Peled E, et al. Reduction of hip joint reaction force via medio-lateral foot center of pressure manipulation in bilateral hip osteoarthritis patients. J Orthop Res. 2016. 10.1002/jor.23190 26865531

[35] Debbi EM, Wolf A, Haim A (2012). Detecting and quantifying global instability during a dynamic task using kinetic and kinematic gait parameters. J Biomech.

[36] Pham T, van der Heijde D, Altman RD, Anderson JJ, Bellamy N, Hochberg M (2004). OMERACT-OARSI initiative: Osteoarthritis Research Society International set of responder criteria for osteoarthritis clinical trials revisited. Osteoarthritis Cartilage.

[37] Angst F, Aeschlimann A, Stucki G (2001). Smallest detectable and minimal clinically important differences of rehabilitation intervention with their implications for required sample sizes using WOMAC and SF-36 quality of life measurement instruments in patients with osteoarthritis of the lower extremities. Arthritis Rheum.

[38] Assa T, Elbaz A, Mor A, Chechik O, Morag G, Salai M (2013). Gait metric profile of 157 patients suffering from anterior knee pain. A controlled study. Knee.

[39] Elbaz A, Mor A, Segal O, Agar G, Halperin N, Haim A (2012). Can single limb support objectively assess the functional severity of knee osteoarthritis?. Knee.

[40] Gigi R, Haim A, Luger E, Segal G, Melamed E, Beer Y (2015). Deviations in gait metrics in patients with chronic ankle instability: a case control study. J Foot Ankle Res.

[41] Khashan M, Mor A, Beer Y, Rath E, Morgensteren DR, Debi R (2014). Gait metric profile and gender differences in hip osteoarthritis patients. A case-controlled study. Hip Int.

[42] Shakoor N, Dua A, Thorp LE, Mikolaitis RA, Wimmer MA, Foucher KC (2011). Asymmetric loading and bone mineral density at the asymptomatic knees of patients with unilateral hip osteoarthritis. Arthritis Rheum.

[43] Studenski S, Perera S, Patel K, Rosano C, Faulkner K, Inzitari M (2011). Gait speed and survival in older adults. JAMA.

[44] Cohen J (1992). A power primer. Psychol Bull.

[45] Durlak JA (2009). How to select, calculate, and interpret effect sizes. J Pediatr Psychol.

[46] Elbaz A, Cohen M, Debbi E, Rath U, Mor A, Morag G (2014). A noninvasive biomechanical treatment as an additional tool in the rehabilitation of an acure anterio cruciate ligamnet tear: a case report. SAGE Open Medical Case Report..

